# Testing as Prevention of Resistance in Bacteria Causing Sexually Transmitted Infections—A Population-Based Model for Germany

**DOI:** 10.3390/antibiotics10080929

**Published:** 2021-07-30

**Authors:** Andreas Hahn, Hagen Frickmann, Ulrike Loderstädt

**Affiliations:** 1 Institute for Medical Microbiology, Virology and Hygiene, University Medicine Rostock, 18057 Rostock, Germany; andreas.hahn@uni-rostock.de (A.H.); or or frickmann@bnitm.de (H.F.); 2 Department of Microbiology and Hospital Hygiene, Bundeswehr Hospital Hamburg, 20359 Hamburg, Germany; 3 Institute for Infection Control and Infectious Diseases, University Medical Center Göttingen, 37075 Göttingen, Germany

**Keywords:** resistance, sexually transmitted infection, prevention, testing, modelling, *Neisseria gonorrhoeae*, *Chlamydia trachomatis*, *Mycoplasma genitalium*

## Abstract

Prescribed antibiotic treatments which do not match the therapeutic requirements of potentially co-existing undetected sexually transmitted infections (STIs) can facilitate the selection of antibiotic-drug-resistant clones. To reduce this risk, this modelling assessed the potential applicability of reliable rapid molecular test assays targeting bacterial STI prior to the prescription of antibiotic drugs. The modelling was based on the prevalence of three bacterial STIs in German heterosexual and men-having-sex-with-men (MSM) populations, as well as on reported test characteristics of respective assays. In the case of the application of rapid molecular STI assays for screening, the numbers needed to test in order to correctly identify any of the included bacterial STIs ranged from 103 to 104 for the heterosexual population and from 5 to 14 for the MSM population. The number needed to harm—defined as getting a false negative result for any of the STIs and a false positive signal for another one, potentially leading to an even more inappropriate adaptation of antibiotic therapy than without any STI screening—was at least 208,995 for the heterosexuals and 16,977 for the MSM. Therefore, the screening approach may indeed be suitable to avoid unnecessary selective pressure on bacterial causes of sexually transmitted infections.

## 1. Introduction

Resistance in sexually transmitted infections (STI) is an issue of increasing concern, a fact which is well-recognized also by military medicine, a discipline in which STIs play a considerable role [1]. While penicillin resistance in *Neisseria gonorrhoeae* was recognized as a problem during the United States (US) military engagement in South-East Asia as early as the middle of the 1960s [2,3], the resistance problem comprised also cephalosporines and tetracyclines about 20 years later, as reported by military personnel in San Diego [4,5]. Consequently, a multi-national surveillance program on drug-resistance of gonococci was established by the US military [6]. This was done for good reason, as the occurrence of strains with combined resistance against both macrolides and 3rd generation cephalosporins made the antimicrobial therapy of gonorrhea increasingly challenging in the recent years [7].

However, gonococci did not remain the sole bacterial infectious agent causing STI with problematic resistance. The occurrence of *Mycoplasma genitalium* with combined resistance against tetracyclines, macrolides, and fluoroquinolones in the course of the previous 30 years became an issue of therapeutic concern as well [8,9], making the application of less effective therapeutic alternatives, such as pristinamycin, necessary [10,11,12].

Exposure to subtherapeutic doses of antibiotic drugs is known to facilitate the emergence of antimicrobial resistance in bacteria [13,14] by supporting the selection of strains carrying, in part, phylogenetically old resistance genes [15]. In particular, such selective processes are triggered by levels of antimicrobial drugs below the mutant prevention concentration (MPV), which is usually higher than the minimum inhibitory concentration (MIC) and triggers the spreading of bacterial cells with predefined resistance mutations [16]. Persistence of such resistant bacteria depends on their competition index, that is, their ability to compete against wild type organisms if the selecting antibiotic pressure vanishes [17], as is caused, for example, by compensatory mutations [18,19,20].

Subtherapeutic dosages of antibiotic drugs, which can facilitate the selection of antimicrobial resistance, are likely to occur in case of blind therapy of a suspected STI without appropriate microbiological diagnosis, as is still frequently the case [1]. Alternatively, this situation may occur if preexisting, asymptomatic bacterial STIs coincide with off-label application of antibiotics, such as doxycycline for pre-exposure prophylaxis (PrEP) or post-exposure prophylaxis (PEP) approaches as practiced by sexually highly active populations with considerable success regarding the prevention of syphilis and *Chlamydia*-associated urethritis [21,22,23,24]. In addition, single-dose application of azithromycin (e.g., for the treatment of gonococcal or chlamydial urethritis) has been reported to trigger the selection of macrolide resistance in *M. genitalium* [25]. Of course, if physicians apply antibiotics for reasons other than the treatment of an STI, they usually do not test for co-occurring subclinical STIs, the resistance rate of which might be affected by the applied courses of antibiotic drugs.

Asymptomatic infections with gonococci, *C. trachomatis,* or *M. genitalium* are not, however, infrequent events; on the contrary, subclinical infections occur quite often [26]. In Germany, this is particularly true for sexually highly active communities of men-having-sex-with-men (MSM) [27], but young, sexually active heterosexual populations are also considerably affected [28,29].

In this modelling-based study, the number-needed-to-test was calculated for identifying sexually transmitted bacterial co-infections requiring consideration prior to the application of antibiotic drugs based on available prevalence data of STIs in the German population. By doing this, it was estimated whether such an approach might be useful for the prevention of resistance selection by the inclusion of co-existing bacterial STIs in planning of the chosen antibiotic therapy.

## 2. Results

For the modelling, the assumptions on the prevalence of bacterial STI, divided into the heterosexual and the MSM (men-having-sex-with-men) population, as well as test characteristics, as detailed in the Materials and Methods section, were applied. A short summary of those assumptions is provided in Table 1, and their background is detailed in the methods chapter.

Based on those assumptions, the predictive values for the screening application of respective diagnostic tests were calculated as shown in Table 2. Thereby, excellent negative predictive values > 99% were seen for all test assays and screened populations. However, while acceptable positive predictive values > 90% were seen for the MSM subpopulation for all assessed bacterial pathogens, the reliability of positive results for *C. trachomatis* and *M. genitalium* in the case of general screenings of the heterosexual population were in the range of tossing a coin. Based on the assumed specificity of the *C. trachomatis* and *M. genitalium* assays, minimum prevalence of 5.27% and 6.16%, respectively, would be necessary to achieve positive predictive values > 90%.

Based on the abovementioned prerequisites, the numbers needed to test to correctly identify an assessed bacterial STI varies from 103 to 104 for the heterosexual population and from 5 to 14 for the MSM population in the case of a screening application. Numbers needed to test for the three different assessed STIs are provided in Table 3. In contrast, for each 144 to 1010 tests for the heterosexual population and each 177 to 1174 tests for the MSM population, a false positive result for bacterial STI is recorded. For the three different assessed STIs, the respective numbers of tests until a false positive result has to be expected are provided in Table 3.

A false positive result may lead to an unnecessary course of antibiotic treatment but not necessarily to a harmful effect on the tested individual. If, however, a false positive result for one bacterial STI meets with a false negative result for another bacterial STI, a potentially inappropriate course of antibiotic treatment for the false bacterial STI may result in additional treatment-associated selective pressure, potentially facilitating resistance selection in the missed bacterial pathogen. The number of screening tests required to meet this undesired situation is defined as the number needed to harm. The number needed to harm for the three assessed bacterial STIs is at least 208,995 for the heterosexual population and 16,977 for the MSM population. The numbers needed to harm stratified by each of the three bacterial STI are shown in Table 3.

To test the robustness of the model, the prevalence rate limits were calculated for which the conclusions of the model were still valid regarding high predictive values with PPV ≥ 0.90 and NPV ≥ 0.99. In detail, a prevalence rate between 0.93% and 20.15% for *N. gonorrhoeae*, 5.27% and 25.08% for *C. trachomatis,* as well as 6.16% and 20.05% for *M. genitalium* are associated with diagnostic PPV ≥ 0.90 and NPV ≥ 0.99 for the given diagnostic sensitivities and specificities of the applied test assays (Table 1). In settings within these prevalence rate limits, preventive screening will lead to reliable diagnostic results. For a further analysis of the limits of our model, the number needed to test for a true positive result, NNT_tp,_ and the number needed to harm, NNH, as defined above were assessed. Thereby, the aim NNT_tp_ < NNH can be considered as ensured based on Equations (6) and (13), if:

$${\mathrm{TNR}}_{k}{\mathrm{TNR}}_{l}\left(1-{\mathrm{NPV}}_{k}{\mathrm{NPV}}_{l}\right)<\frac{{\mathrm{PPV}}_{i}}{\left(1-{\mathrm{PPV}}_{i}\right)}$$

This, however, even holds for PPV_i_, NPV_k_, and NPV_l_ ≥ 0.5, which can be assumed as an absolute minimum for predictive values of any diagnostic assay to be accepted for diagnostic purposes.

## 3. Discussion

It is a well-established paradigm of infectious disease medicine that many molecular resistance mechanisms are ancient and highly conserved [15], while selective pressure due to inappropriate antibiotic therapy facilitates the selection of pre-existing isolates harboring molecular resistance determinants [13,14,16]. While inappropriate antimicrobial therapy (e.g., due to suboptimal choice of antimicrobial drugs or due to subtherapeutic and thus resistance-selecting dosage) is usually not intended, it may easily occur if the respective infection is unknown due to a subclinical course, a problem which has been extensively discussed during the roll-out of HIV (human immunodeficiency virus) PrEP [30]. As, for example, stated in the German-Austrian HIV-PrEP guideline [31], it makes sense to exclude prevalent infection prior to prophylactic use of antimicrobial drug application in order to prevent selection of antimicrobial resistance. Other than antibiotic drugs, however, anti-retroviral drugs are usually not applied, apart from HIV-specific therapy, HIV-PEP, and HIV-PrEP, so the risk of resistance selection by chance is quite low. Here, we have performed a modelling on the potential usefulness of screenings for bacterial STI prior to antibiotic therapy for any reason, using the example of the epidemiological situation in Germany.

Other than anti-retroviral drugs, antibiotics are frequently applied in spite of uncertainty regarding the co-occurrence of asymptomatic bacterial STIs in individuals under therapy due to their lacking a diagnosis. This could contribute to the ongoing selection of resistant bacterial agents causing STIs. The here-described modelling based on the prevalence rates in Germany suggested that screening for STIs may be a strategy to prevent resistance in bacteria causing sexually transmitted infections, because numbers needed to test were considerably lower than numbers needed to harm.

In this modelling, “harm” was defined as missing an existing bacterial STI and receiving a false positive result for another one instead as a consequence of imperfect test accuracy, with the associated risk of inappropriate and thus potentially resistance-selecting therapy. In spite of higher numbers needed to test in the heterosexual compared to the MSM population, the numbers needed to harm were considerably higher among heterosexuals than in the MSM group. This effect was due to the low prevalence among heterosexuals. Accordingly, the probability of an unfortunate combination of test results potentially triggering an inappropriate adoption of antibiotic therapy was very low for both populations assessed in the applied model.

The modelling conducted in this study has been newly designed by the authors for this academic question. The underlying mathematic paradigms are, however, not new. They comprise the binomial distribution of diagnostic test results, the resulting expected values for positive and negative test results, and Bayes’ theorem, which together provide the mathematical background for the definition of diagnostic sensitivity and specificity, as well as positive and negative predictive values, and which have been described in detail elsewhere [32]. Based on this background, the modelling can be considered as robust and basically suitable to assess numbers needed to test as well as numbers needed to harm for the defined epidemiological settings.

Due to the fact that the assumptions on bacterial STI prevalence in Germany which were used as premises for the modelling imply a degree of uncertainty based on the lacking STI surveillance in Germany, and the resulting need for reliance on prevalence studies, the model was assessed for robustness by calculating the limits for which the conclusions of the model were still valid. Thereby, considerable robustness could be shown, making it likely that the conclusions will hold even if the prevalence assumptions were moderately over- or underestimated. Nevertheless, it is undeniably true that the results as calculated for Germany in the modelling presented here do not necessarily apply for all regional settings, as the German situation was just exemplarily chosen because the authors are familiar with it. If desired, however, the calculations from the model can be applied for other geographical settings as well, as long as sufficient data for prevalence assumptions is available, for example, from surveillance platforms linked by the International Union against Sexually Transmitted Infections (IUSTI) (https://iusti.org/epidemiology/, accessed on 15 July 2021). Thereby, even further differentiation as stratified by the US American Centers for Disease Control and Prevention (CDC) for the *C. trachomatis* prevalence in different US sub-populations [33] can be included as well to allow more precise, sub-population-adapted conclusions on the potential benefits or risks of rapid STI testing prior to intended antibiotic therapies.

Rapid molecular detection of bacterial STI alone can, however, not circumvent all obstacles. Even when bacterial STI are rapidly diagnosed with molecular assays prior to intended antibiotic treatment for other reasons, the problem remains that molecular screening assays usually do not provide susceptibility information required to guide an STI-specific antimicrobial treatment. While susceptibility-guided therapy should remain the gold standard, molecular STI detections will only allow calculated antimicrobial therapy in line with national guidelines, requiring some expertise in the field of STI therapy. Therefore, the British Association for Sexual Health and HIV (BASHH) discourages testing if appropriate follow-up management in case of positive results cannot be ensured, in order to avoid complications resulting from inappropriate therapeutic advice (https://www.bashhguidelines.org/current-guidelines/urethritis-and-cervicitis/bashh-position-statement-on-the-inappropriate-use-of-multiplex-testing-platforms-and-suboptimal-antibiotic-treatment-regimens-for-bacterial-sexually-transmitted-infections/, accessed on 14 July 2021).

Indeed, even calculated therapy of correctly identified bacterial STIs is quite challenging and should occur in line with regionally applicable guidelines considering resistance surveillance data. As stated above, antimicrobial resistance is an issue of increasing concern, as recently exemplified for the history of resistance accumulation in *Neisseria gonorrhoeae* [34]. Even for the treatment of *Neisseria gonorrhoeae*, regional guidelines vary for the dosages of the “standard” therapeutic single dose regiment comprising ceftriaxone and azithromycin between the German guideline (https://www.awmf.org/leitlinien/detail/ll/059-006.html, accessed on 15 July 2021), the BASHH guideline from the United Kingdom [35], the guideline by the IUSTI for Europe [36], and the CDC guideline [37]. For alternative calculated therapy or therapy regimens in case of existing susceptibility testing results, the differences are even bigger. Varying treatment recommendations also exist for the management of other bacterial STIs considering regional susceptibility rates [38,39,40,41,42].

While susceptibility testing in *Neisseria gonorrhoeae* is usually based on growth in culture, molecular methods beyond rapid screening tests are usually necessary to diagnose resistance in obligatorily intracellular bacterial causes of STI [43]. Concerns about antimicrobial resistance, however, are not the only reason for STI screening, which plays an important role in the reduction of transmission of STIs as well as of serious complications of asymptomatic STIs, like pelvic inflammatory disease (PID) in women or epididymitis in men [43].

The study has a number of limitations. First of all, the prevalence values applied are just rough estimations based on cross-sectional assessments [27,28,29], because continuous surveillance of bacterial STIs is not performed in Germany. Second, the modelling ignores that screening and subsequent treatment as well as contemporaneous new infections will also affect the pre-existing prevalence. The inclusion of respective temporary variations in consequence of a hypothetical STI screening prior to antibiotic therapy are, however, affected by multiple factors that are hardly predictable. An example is the “risk compensation” phenomenon mediated by the so-called Peltzmann effect [44] as reported for PrEP-based HIV-prevention [45]. Third, a theoretical modelling cannot definitively ensure a practical impact of a preventive medical approach, requiring a future proof-of-principle study to verify or falsify the hypotheses of the study presented here. Finally, rapid STI testing prior to antibiotic prescriptions by physicians will most likely only affect the intake of antibiotics due to medical indications. Off-label antibiotic application, for example, self-administered postexposure prophylaxis with doxycycline after risky sexual contacts, which is explicitly discouraged by BASHH and Public Health England due to an uncertain risk-to-benefit ratio (https://www.bashhguidelines.org/media/1156/doxy_pep_statement_v5_phe_bashh.pdf, accessed on 15 July 2012), will hardly be influenced by such a strategy.

## 4. Materials and Methods

### 4.1. Prevalence Assumptions

Up-to-date prevalence data for *N. gonorrhoeae*, *C. trachomatis*, and *M. genitalium* in Germany are particularly available for the MSM community. Prevalence in at least one accessible cavity for diagnostic screening (pharynx, anus) ranged from 7.2% to 13.8% for *C. trachomatis*, from 14.2% to 19.4% for *M. genitalium*, and from 7.4% to 14.8% for *N. gonorrhoeae*, respectively [27]. In a recent representative assessment, the proportion of MSM in the German population has been estimated to be round about 4% of the males, and thus about 2% of the total population [46].

For the heterosexual population, the availability of both reliable and representative prevalence data is scarce. Based on a recent meta-analysis, the prevalence of *M. genitalium* in any accessible cavity for screening (pharynx, anus, vagina) can be estimated to be around about 1% [29]. In a recent study on young German adolescents containing a high proportion of MSM among the male study population as well as individuals with high proportions of experience with “risk practices” like anal intercourse, 7.7% were positive for *C. trachomatis* and 5.5% for *N. gonorrhoeae* [28]. Considering the fact that a sexually highly active population was screened, for which number of sexual partners has been estimated to be about three times as high as for the general population for both sexes [46], and also considering the comparably high proportion of MSM among the screened males, it is likely that the true prevalence of *N. gonorrhoeae* and *C. trachomatis* among the general heterosexual population will be round about 1% or lower. Accordingly, prevalence of ≤1% was estimated for *C. trachomatis*, *M. genitalium*, and *N. gonorrhoeae* each for the heterosexual general population in Germany for the modelling.

### 4.2. Test Assay Assumptions

Test characteristics of modern molecular diagnostic assays for *C. trachomatis* and *N. gonorrhoeae* are well established [47,48,49,50]. Accordingly, sensitivity of 97.0% and specificity of 99.4% were assumed for the *C. trachomatis*-specific assay as well as sensitivity of 96.0% and specificity of 99.9% for the *N. gonorrhoeae*-specific assay, respectively [50]. For rapid molecular screening for *M. genitalium*, however, the amount of available data on diagnostic accuracy is smaller. A recent validation [51] indicated a limit-of-detection of 10 copies, which is in a similar range as described for *N. gonorrhoeae*-specific PCR, so sensitivity of 96.0% as reported for the *N. gonorrhoeae*-specific assay is also assumed for the *M. genitalium*-specific assay in the modelling. Regarding specificity, the situation is more complex, as it is challenging to discriminate true from false positive results due to the lack of a reference standard in the study [51]. In the study [51], 8 out of 295 samples from a high-risk population tested positive for *M. genitalium*. Assuming a prevalence of about 1% [29] even in the standard population, between 0 and 2 of the assessed samples are likely to have been false positives, resulting in specificity ≥ 99.3%. Based on those considerations, sensitivity of 96.0% and specificity of 99.3% was assumed for molecular testing for *M. genitalium*.

### 4.3. Modelling Approach

Based on the binomial distribution of diagnostic test results with given diagnostic sensitivity Se, diagnostic specificity Sp, and prevalence rate Prev, expected values for the rates of positives and negatives in diagnostic assays (TPR and TNR) are given by:

(1) TPR=Se*Prev+(1−Sp)(1−Prev)

(2) TNR=Sp(1−Prev)+(1−Se)Prev=1−TPR

Positive and negative predictive values (PPV and NPV) are given by:

(3) $$\mathrm{PPV}=\frac{\mathrm{Se}*\mathrm{Prev}}{\mathrm{Se}*\mathrm{Prev}+\left(1-\mathrm{Sp}\right)\left(1-\mathrm{Prev}\right)}$$

(4) $$\mathrm{NPV}=\frac{\mathrm{Sp}\left(1-\mathrm{Prev}\right)}{\mathrm{Sp}\left(1-\mathrm{Prev}\right)+\left(1-\mathrm{Se}\right)\mathrm{Prev}}$$

The number needed to test for a positive test result NNT_p_ is given by:

(5) $${\mathrm{NNT}}_{p}=\frac{1}{\mathrm{TPR}}$$

The number needed to test for a true positive test result NNT_tp_ is given by:

(6) $${\mathrm{NNT}}_{\mathrm{tp}}=\frac{1}{\mathrm{TPR}*\mathrm{PPV}}$$

The number needed to test for a false positive test result NNT_fp_ is given by:

(7) $${\mathrm{NNT}}_{\mathrm{fp}}=\frac{1}{\mathrm{TPR}*\left(1-\mathrm{PPV}\right)}$$

The number needed to test for a negative test result NNT_p_ is given by:

(8) $${\mathrm{NNT}}_{n}=\frac{1}{1-\mathrm{TPR}}$$

The number needed to test for a true negative test result NNT_tn_ is given by:

(9) $${\mathrm{NNT}}_{\mathrm{tn}}=\frac{1}{\left(1-\mathrm{TPR}\right)*\mathrm{NPV}}$$

The number needed to test for a false negative test result NNT_fn_ is given by:

(10) $${\mathrm{NNT}}_{\mathrm{fn}}=\frac{1}{\left(1-\mathrm{TPR}\right)*\left(1-\mathrm{NPV}\right)}$$

Considering a positive predictive value PPV as desired, the minimum prevalence rate to reach this PPV is given by:

(11) $$\mathrm{Prev}=\frac{1-\mathrm{Sp}}{\frac{\mathrm{Se}}{\mathrm{PPV}}-\mathrm{Se}-\mathrm{Sp}+1}$$

If a defined positive predictive value NPV is desired, the maximum prevalence rate to reach this NPV is given by:

(12) $$\mathrm{Prev}=\frac{\mathrm{Sp}}{\frac{\left(1-\mathrm{Se}\right)\mathrm{NPV}}{1-\mathrm{NPV}}+\mathrm{Sp}}$$

The number needed to harm NNH as defined above is given by:

(13) $$\mathrm{NNH}=\frac{1}{{\mathrm{TPR}}_{i}\left(1-{\mathrm{PPV}}_{i}\right){\mathrm{TNR}}_{k}{*\mathrm{TNR}}_{l}\left(1-{\mathrm{NPV}}_{k}{*\mathrm{NPV}}_{l}\right)}$$

Thereby, i, k, and l represent the three STIs caused by *N. gonorrhoeae*, *C. trachomatis*, and *M. genitalium*.

## 5. Conclusions

In spite of the abovementioned limitations, the modeling showed that screening for asymptomatic STIs based on rapid molecular diagnostic assays prior to antibiotic therapy may be a promising strategy to prevent resistance selection in bacteria causing sexually transmitted infections. Real world assessments including the use of rapid molecular STI screening assays are required to confirm the results of the model as well as the practicability of the approach.

## Figures and Tables

**Table 1:** Assumptions regarding STI prevalence and test accuracy.

	*N. gonorrhoeae*	*C. trachomatis*	*M. genitalium*
Prevalence Rate (Heterosexuals)	1%	1%	1%
Prevalence Rate (MSM)	7.4% to 14.8%	7.2% to 13.8%	14.2% to 19.4%
Sensitivity	0.960	0.970	0.960
Specificity	0.999	0.994	0.993

MSM = men-having-sex-with-men.

**Table 2:** Predictive values of screening tests based on the assumptions from Table 1.

	*N. gonorrhoeae*	*C. trachomatis*	*M. genitalium*
PPV (Heterosexuals)	0.9065	0.6202	0.5808
NPV (Heterosexuals)	0.9996	0.9997	0.9996
PPV (MSM)	0.9871 to 0.9940	0.9262 to 0.9628	0.9578 to 0.9706
NPV (MSM)	0.9931 to 0.9968	0.9952 to 0.9977	0.9904 to 0.9934
Minimum prevalence needed to achieve PPV ≥ 90%	0.93%	5.27%	6.16%
Maximum prevalence needed to achieve NPV ≥ 99%	20.15%	25.08%	20.05%

PPV = positive predictive value. NPV = negative predictive value. MSM = men-having-sex-with-men.

**Table 3:** Numbers needed to test (NNT) for a true positive test result per species, expected numbers of tests per species until a false positive result is seen, and species-specific numbers needed to harm (NNH) as defined by false positive results in the species-specific screening assay combined with false negative results for at least one of the other bacterial STIs.

	*N. gonorrhoeae*	*C. trachomatis*	*M. genitalium*
NNT for a true positive test result (heterosexuals)	104	103	104
NNT for a true positive test result (MSM)	7 to 14	7 to 14	5 to 7
NNT for a false positive test result (heterosexuals)	1010	168	144
NNT for a false positive test result (MSM)	1080 to 1174	180 to 193	167 to 177
NNT for a true negative test result (heterosexuals)	1.01	1.02	1.02
NNT for a true negative test result (MSM)	1.08 to 1.17	1.08 to 1.17	1.17 to 1.25
NNT for a false negative test result (heterosexuals)	2500	3333	2500
NNT for a false negative test result (MSM)	169 to 338	242 to 463	129 to 176
NNT for a positive test result (heterosexuals)	94.43	63.94	60.50
NNT for a positive test result (MSM)	7.00 to 13.90	7.19 to 13.26	5.21 to 7.03
NNT for a negative test result (heterosexuals)	1.01	1.02	1.02
NNT for a negative test result (MSM)	1.08 to 1.17	1.08 to 1.16	1.17 to 1.24
Expected positive test rate (heterosexuals)	0.011	0.016	0.017
Expected positive test rate (MSM)	0.072 to 0.143	0.075 to 0.139	0.142 to 0.192
NNH as defined by a false positive result combined with false negative results for at least one of the other bacterial STIs (heterosexuals)	1,466,752	213,374	208,995
NNH as defined by a false positive result combined with false negative results for at least one of the other bacterial STIs (MSM)	117,434 to 152,272	16,977 to 23,046	20,560 to 35,164

NNT = number needed to test. NNH = number needed to harm. MSM = men-having-sex-with-men.

## Data Availability

Not applicable.
